# Does cognitive flexibility predict treatment gains in Internet-delivered psychological treatment of social anxiety disorder, depression, or tinnitus?

**DOI:** 10.7717/peerj.1934

**Published:** 2016-04-18

**Authors:** Philip Lindner, Per Carlbring, Erik Flodman, Amanda Hebert, Stephanie Poysti, Filip Hagkvist, Robert Johansson, Vendela Zetterqvist Westin, Thomas Berger, Gerhard Andersson

**Affiliations:** 1 Department of Psychology, Stockholm University, Stockholm, Sweden; 2 Department of Clinical Neuroscience, Karolinska Institute, Stockholm, Sweden; 3 Department of Behavioural Sciences and Learning, Linköping University, Linköping, Sweden; 4 NeuroVux Clinic, Norrbotten County Council, Luleå, Sweden; 5 Department of Neuroscience, Uppsala University, Uppsala, Sweden; 6 Department of Clinical Psychology and Psychotherapy, University of Bern, Bern, Switzerland

**Keywords:** Wisconsin Card Sorting Test, Perseveration, Prediction, Psychotherapy, Internet

## Abstract

Little is known about the individual factors that predict outcomes in Internet-administered psychological treatments. We hypothesized that greater cognitive flexibility (i.e. the ability to simultaneously consider several concepts and tasks and switch effortlessly between them in response to changes in environmental contingencies) would provide a better foundation for learning and employing the cognitive restructuring techniques taught and exercised in therapy, leading to greater treatment gains. Participants in three trials featuring Internet-administered psychological treatments for depression (n = 36), social anxiety disorder (n = 115) and tinnitus (n = 53) completed the 64-card Wisconsin Card Sorting Test (WCST) prior to treatment. We found no significant associations between perseverative errors on the WCST and treatment gains in any group. We also found low accuracy in the classification of treatment responders. We conclude that lower cognitive flexibility, as captured by perseverative errors on the WCST, should not impede successful outcomes in Internet-delivered psychological treatments.

## Introduction

Mounting evidence suggests that Internet-delivered psychological treatment programs based on cognitive behavioral therapy (CBT) (Andersson, 2009; Andersson, 2014) are effective treatments of mood disorders, anxiety disorders, and somatic disorders like tinnitus (Andersson et al., 2013). More recently, similar treatment programs based on psychodynamic principles have also been created and found to be effective (Johansson et al., 2012). Although several studies have investigated predictors of treatment response in Internet interventions (Nordgreen et al., 2012; Hedman et al., 2012), few studies (Andersson, Carlbring & Grimlund, 2008) have investigated the impact of individual differences in executive functions on treatment gains.

Cognitive flexibility (CF), an executive function, refers to the ability to simultaneously consider several concepts and tasks, and switch effortlessly between them in response to changes in environmental contingencies (Miyake et al., 2000). The conceptualization of CF is similar to the processes involved in the cognitive restructuring techniques used in CBT. In cognitive restructuring, the patient is taught to identify negative automatic thoughts, find ways to reality-test these thoughts, and then develop novel, more functional ways of interpreting the situation (Johnco, Wuthrich & Rapee, 2014). This conceptual overlap between CF and cognitive restructuring techniques suggests that pre-treatment CF abilities may predict the successful acquisition and use of CF strategies in CBT, leading to greater treatment gains. Lower CF abilities have often been associated with mental disorders, especially depression (Snyder, Miyake & Hankin, 2015). Emerging findings have also implicated abnormal cognitive processes in tinnitus (Andersson & McKenna, 2006), and cognitive restructuring is a key part of CBT treatment of this condition (Hesser et al., 2012). Despite these findings associating CF and mental distress, there is limited research on whether CF predicts psychotherapy outcomes for mental disorders, and the few findings that are available have been mixed (Mohlman & Gorman, 2005; Moritz et al., 2005; D’Alcante et al., 2012). One previous study found an association between cognitive flexibility and cognitive restructuring skill acquisition (Johnco, Wuthrich & Rapee, 2013), while another failed to find such an association (Johnco, Wuthrich & Rapee, 2014).

No previous study has investigated whether CF predicts outcomes in Internet-delivered CBT, which despite sharing many similarities with traditional CBT, has some inherent characteristics that we reasoned should put a greater demand on CF abilities. In most Internet interventions, psychoeducation, cognitive and behavioral exercises and other therapeutic components cannot be customized by a therapist according to the patient’s unique situation to the same degree as in traditional therapy. Few Internet interventions feature live therapist contact, meaning that it is not possible for the therapist and the patient to work together on a cognitive restructuring task. Hence, successfully completing Internet interventions likely requires a greater ability to not only comprehend the delivered therapeutic material, but also to identify emotional, cognitive and behavioral patterns entirely independently or with very little therapist assistance. Although cognitive restructuring has traditionally been considered a therapeutic technique specific to CBT, CF may also be important in psychodynamic therapy, where the goal is to explore, identify and change unconscious patterns of cognitions and emotions (Johansson et al., 2012). Little research has investigated neurocognitive predictors of treatment response to psychodynamic therapy, and there has been no study on CF and Internet-administered psychodynamic therapy.

The 64-card Wisconsin Card Sorting Test (WCST) (Greve, 2001) is an established neuropsychological test of set-shifting ability, i.e. CF in the face of changing reinforcement schedules (Dehaene & Changeux, 1991). We reasoned that greater CF prior to commencing therapy (as measured by a low number of perseverative error on the WCST) should provide a better foundation for learning, improving and utilizing strategies that rely on CF taught in therapy, regardless of type of therapy, leading to greater symptom reduction. If such an association were to be found, it would justify efforts to personalize clinical treatments based on the patient’s neuropsychological abilities to achieve better outcomes. Studying the predictive power of CF in the context of psychotherapy also provides indirect insight into the mechanisms of therapeutic change. Here we report the results of a multi-sample study designed to investigate the association between CF and symptom reduction in Internet-delivered psychological treatment.

## Materials and Methods

Data for the current study was collected as part of three randomized trials: therapist-guided CBT for social anxiety disorder (SAD) (Boettcher et al., 2014), psychodynamic treatment for major depressive disorder (MDD) (Johansson et al., 2012) and CBT or Acceptance and Commitment Therapy (ACT) for tinnitus (Hesser et al., 2012), all Internet-delivered. The primary results of these studies have been reported previously; for full methods, see the respective publication. The intervention studies received ethical approval from the regional ethical review boards in either Linköping (2010/386-31 and 2008/234-08) or Umeå (2012/132-31Ö), and all participants provided written, informed consent.

### Samples

Because the aim of the current study was not to evaluate treatment efficacy, analyses were conducted per protocol: participants classified as treatment dropouts (including those with zero completed treatment modules) and/or had incomplete post-treatment data were not included in analyses. One additional participant in the SAD group with a WCST total completion time of 5170 s (final sample mean = 407.72 [SD = 151.51]) was excluded due to presumed technical issues with the online WCST administration. Final sample sizes for the SAD group was n = 115 (all received treatment), n = 83 for the tinnitus group (n = 53 received treatment: n = 28 ACT, n = 25 CBT), and n = 73 for the MDD group (n = 36 received treatment). Pre- and post-treatment symptom scores on the primary outcome measure in each study, along with WCST metrics, age and sex for each subject, were compiled (see Table 1). The total sample mean age was 41.65 (SD = 14.66) years, but there was an overall difference in age (*F*[2,268] = 35.18, *p* < .001), driven by SAD < MDD (*p* < .001) and SAD < tinnitus (*p* < .001) pair-wise differences (Bonferroni-corrected *p* < .05). Groups were not matched on sex (Fisher’s exact test *p* = .0014), but because there was no sex-difference in any WCST score (all *p* > .157), this was not considered a confounding variable. On raw WCST metrics, groups differed only on Total completion time; however, there were no differences between groups in age-adjusted WCST metrics (all *F*[2,268] < 1.21, *p* > .3).

**Table 1:** Sample characteristics.

Variable	A. Social anxiety disorder	B. Tinnitus	C. Depression	Statistics
Mean age (SD)	34.09 (10.53)	49.13 (14.94)	45.04 (14.44)	*F*[2,268] = 35.18, *p* < .001, A < (B, C)
% Females	60.9%	47.0%	75.3%	FET *p* = .0014
Perseverative errors†	13.35 (6.08)	14.39 (6.05)	13.92 (6.93)	*F*[2,268] = 0.663, *p* = .516
Total errors†	20.14 (8.31)	22 (9.04)	22.22 (10.15)	*F*[2,268] = 1.566, *p* = .211
Categories completed†	2.44 (1.42)	2.46 (1.44)	2.33 (1.47)	*F*[2,268] = 0.189, *p* = .828
Total correct†	43.86 (8.31)	42 (9.04)	41.78 (10.15)	*F*[2,268] = 1.566, *p* = .211
Trials to complete first category†	19.17 (12.55)	20.12 (13.78)	22.11 (14.45)	*F*[2,268] = 1.074, *p* = .343
Completion time†	367.59 (114.45)	453.84 (152.5)	418.51 (183.62)	*F*[2,268] = 8.15, *p* < .001, A < B*

**Notes:**

† Raw scores, not adjusted for age.

* No longer significant when using age-adjusted completion time. FET, Fisher’s exact test.

### Procedures

In the MDD and tinnitus samples, WCST and pre-treatment (screening) data was collected prior to any treatment commencing, and post-treatment data soon afterwards. In the SAD sample, WCST data and pre-treatment symptom scores were collected after two weeks of initial attention bias modification, and post-treatment symptoms scores after a nine-week CBT program. The two forms of attention bias modification used produced similar results (Boettcher et al., 2014), and were hence considered equal in the current study. To account for the possibility that CF was associated with treatment gains only in certain investigated treatment types, analyses were conducted both using the full sample and group-wise.

### Measures

#### Online Wisconsin Card Sorting Test

**Figure 1:**
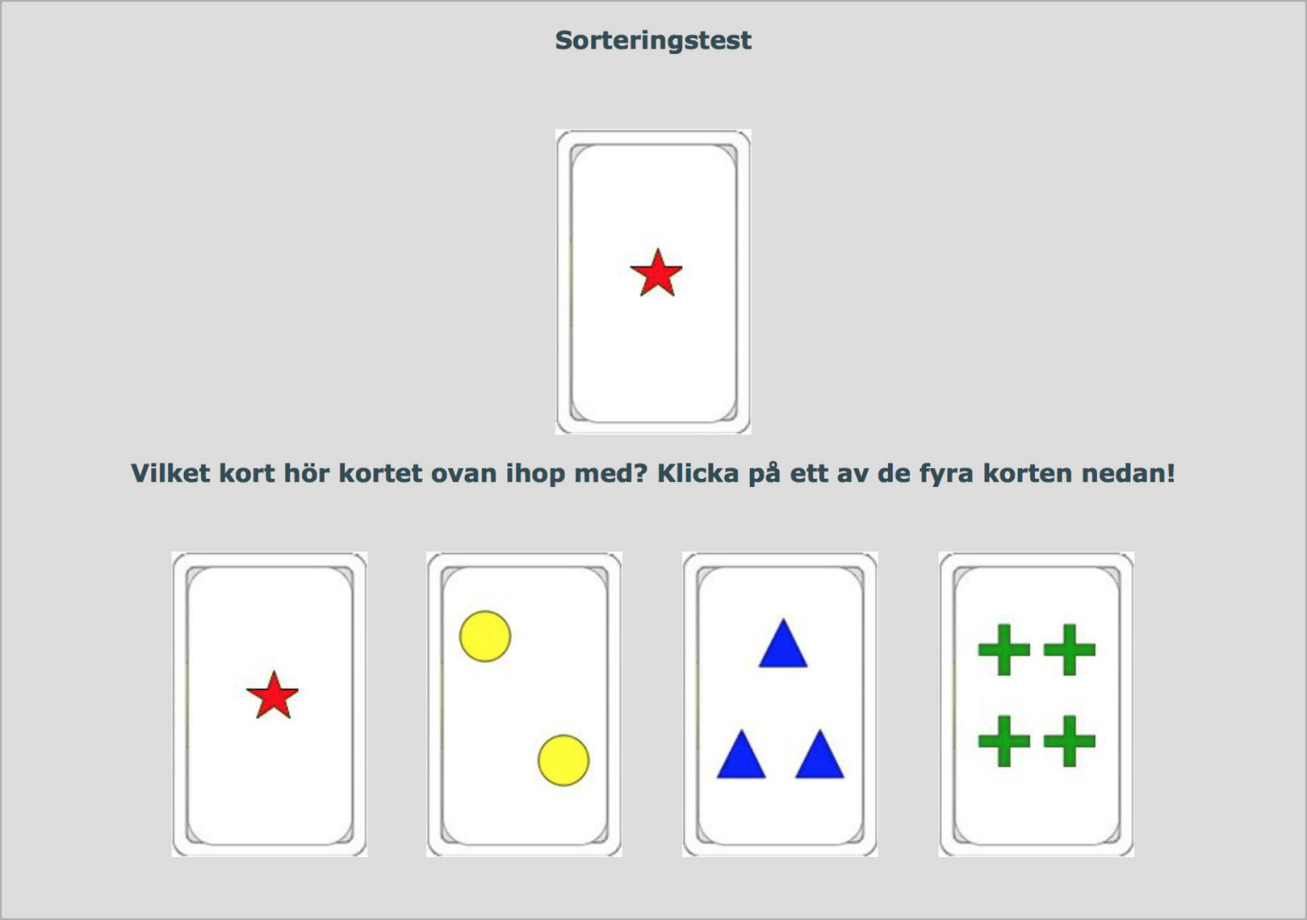
Screenshot of the online WCST. Translation from Swedish: “Sorting test. Which card does the card above belong with? Click on one of the four cards below!”

An online, in-house developed version of the standard, single-deck version of the WCST (Greve, 2001) featuring 64 response cards, each administered together with four stimulus cards, was completed by all participants prior to commencing treatment (see Fig. 1 for screenshot). Previous research (Axelrod, Paolo & Abraham, 1997) has shown that in clinical settings it is inappropriate to extrapolate norms from the full WCST version to the single-deck version used in the current study. In the WCST, participants are tasked with sorting the response card by matching it to the stimulus card. They are not told about the matching principle (number, color or shape), but are forced to deduce this based upon the feedback received after the last sorting attempt. Number of Perseverative errors (PE), i.e. the continued used of a sorting strategy that was labelled incorrect during the last trial, was used as CF metric. Clinical WCST norm data are age-adjusted, but because the validity of applying foreign, pen-and-paper WCST norms to our online version has not been independently validated, we opted instead to take age into account by regressing out the effect of age on number of PE and using this age-adjusted metric for statistical analyses. Since the current study did not feature a representative, healthy comparison group, percentage PE of total errors was also calculated for each subject and regression analyses repeated using these self-normalized scores.

We assessed the psychometric validity of online WCST by performing a principal component analysis (PCA) to estimate the latent structure of our results and comparing with past findings. A composite two-factor structure has been proposed, derived from a meta-analysis of ten factor analyses in four studies featuring neurological, senior and/or healthy samples (Greve, Ingram & Bianchini, 1998). The proposed composite solution includes Categories completed and Total correct scores loading oppositely to Perseverative errors and Total errors on factor one, with zero loadings on factor two for all but Total correct. Due to unavailable WCST metrics, we were unable to replicate the exact analysis. This notwithstanding, performing a principal component analysis with forced two-component extraction resulted in a structure generally consistent with the proposed composite two-factor solution with regards to component direction and strength. This was despite substantial differences in sample characteristics, suggesting adequate psychometric validity (see Table S1 for more details).

#### Symptom measures

The primary outcome measures in the studies were the Liebowitz Social Anxiety Scale—Self-Report version (Fresco et al., 2001), the Beck Depression Inventory II (Beck, Steer & Brown, 1996), and the Tinnitus Handicap Inventory (Newman, Jacobson & Spitzer, 1996). Raw pre-post score differences, along with percentage symptom reduction (PSR) (D’Alcante et al., 2012) in participants who received active treatment (total n = 204) was calculated. The latter was calculated to provide comparable cross-sample scores. PSR differed between groups [*F*[2,201] = 15.03, *p* < .001), such that PSR_MDD_ > PSR_Tinnitus_ > PSR_SAD_ (all pair-wise contrast *p* < 0.027, Bonferroni-corrected).

### Statistical analyses

We used two methods to test our hypothesis that cognitive flexibility is associated with treatment gains. First, age-adjusted PE was entered as a predictor of raw pre-treatment symptom scores in standard linear regression models, group-wise. Then, number of PE was used to predict PSR in the full sample and group-wise, and pre-post raw score difference group-wise. These analyses were repeated featuring percentage PE of total errors.

Second, performance of the PE metric in classifying treatment responders vs. low-responders was investigated using receiver-operating characteristics (ROC). Symptom reductions of 25%, 50% and 75% were used as separate thresholds for treatment response. Analyses were conducted both group-wise and featuring the full sample. Bootstrapped 95% confidence intervals for area under the curve (AUC) for each classification curve were used to test whether classification accuracy differed from chance. All analyses were conducted in the R (3.2) statistical environment, using the pROC (Robin et al., 2011) and psych (Revelle, 2015) packages.

## Results

### Linear associations

**Figure 2:**
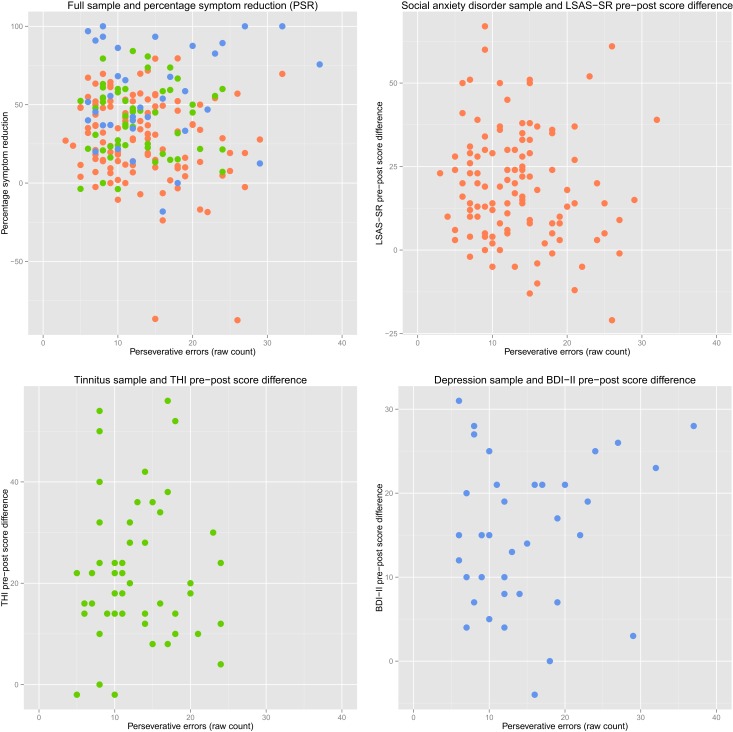
Perseverative errors and symptom reduction scatterplots. Red scatter: Social anxiety disorder sample. Green scatter: Tinnitus sample. Blue scatter: Depression sample. Plotted PE scores are not age-adjusted.

There were no significant associations between pre-treatment symptom scores and PE in any group. There were also no significant associations between PE and pre-post difference in symptom scores in any group, or any significant associations between PE and PSR in either the full sample or each group separately (see Table 2). In the SAD group, the positive association between PSR and PE approached significance (*p* = .065). However, inspection of the scatter plot revealed two PSR outliers more than three standard deviations from the group mean. If these were removed, the correlation was no longer approaching significance (see Fig. 2 for scatterplots). Re-running all analyses using percentage PE of total errors did not change results.

**Table 2:** Univariate association between perseverative errors and treatment gains.

Sample	n	Outcome	Parameter estimate	Parameter 95% CI	*p*
Pre-treatment scores					
Social anxiety disorder	115	LSAS-SR	−0.145	−0.804—0.513	0.663
Tinnitus	83	THI	0.104	−0.437—0.645	0.703
Depression sample	73	BDI-II	0.051	−0.167—0.270	0.640
Pre-post score difference					
Social anxiety disorder	115	LSAS-SR	−0.284	−0.800—0.232	0.278
Tinnitus	53	THI	0.145	−0.602—0.892	0.699
Depression sample	36	BDI-II	0.162	−0.224—0.549	0.399
Pre-post Percentage Symptom Reduction (PSR)					
Full sample	204	PSR	−0.233	−0.876 —0.410	0.476
Social anxiety disorder	115	PSR	−0.790†	−1.629—0.049†	0.065†
Tinnitus	53	PSR	0.148	−1.081—1.376	0.811
Depression sample	36	PSR	0.495	−0.868—1.858	0.466

**Note:**

† No longer approaching significance (*p* > .075) after removal of two PSR outliers. Perseverative errors are age-adjusted.

### Classification accuracy

**Figure 3:**
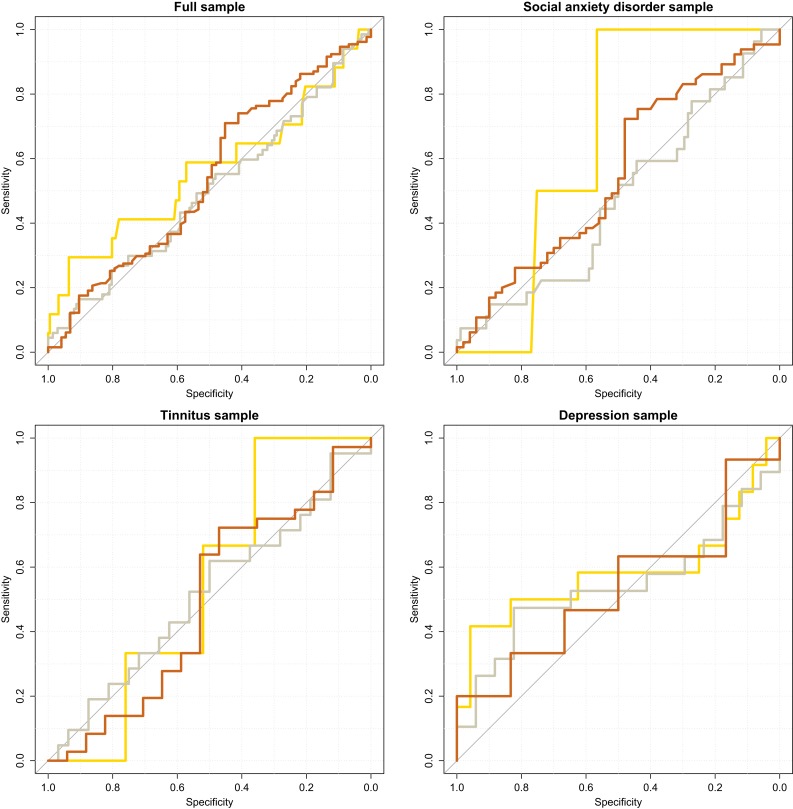
Accuracy of perseverative errors in classifying treatment improvers. Lines colored according to treatment response thresholds. Gold: 75% Percentage Symptom Reduction (PSR). Silver: 50% PSR. Bronze: 25% PSR. Age-adjusted PE scores used.

Out of the twelve ROC curves investigated (full sample + group-wise × three thresholds for treatment response), there was only one instance when the AUC confidence interval did not cover 0.5. In this case, in the SAD group using the 75% PSR cut-off, only n = 2 scored above the PSR cut-off, leading to an irregular curve shape and a confidence interval ranging 0.51–0.81 (see Fig. 3 for ROC curves).

## Discussion

Contrary to our hypothesis, we found no evidence suggesting an association between our measure of pre-treatment CF and post-treatment improvement. This indicates that comparably low CF (measured by number of perseverative errors on the WCST) should not be an obstacle in achieving symptom reduction after Internet-delivered psychological treatment, and that pre-treatment measurements of CF will not predict treatment outcomes. The lack of predictive power is congruent with there being no linear correlation between pre-treatment symptom measures and number of PE.

In the context of traditional psychotherapy, findings on associations between CF and cognitive restructuring skill acquisition, considered important for improvement, have been mixed (Johnco, Wuthrich & Rapee, 2013; Johnco, Wuthrich & Rapee, 2014). We reasoned that because Internet-delivered therapy by design includes less contact with a therapist (if included at all), it requires the patient to have greater CF abilities to independently identify cognitive, behavioral and emotional patterns, and to successfully apply cognitive restructuring techniques to improve their condition. While our study design cannot directly test this assumption, our negative finding, consistent with some prior research on traditional psychotherapy, suggests that even in psychotherapeutic formats that place a greater demand on the patient’s own ability to comprehend and successfully execute cognitive restructuring exercises, there is no association between CF (as measured by the WCST) and treatment gains.

While lower CF is often reported in psychiatric samples, comparably little is known about the precise relationship between CF and psychiatric symptoms. It is noteworthy that even direct training of executive functions (including CF) appears to have little or no generalized effect beyond improved performance on the specific task trained (Snyder, Miyake & Hankin, 2015). Recently, variants of the WCST that feature emotional content (Deveney & Deldin, 2006; Aker & Landrø, 2014) have been developed and validated. Future studies should investigate whether these have an improved ability to differentiate clinical from non-clinical samples and predict psychotherapy outcomes. Future studies featuring larger samples should measure a wider range of executive functions, such as response inhibition and set-updating, and preferably in an affective context. This will likely be required in order to accurately model the complex neurocognitive mechanisms implicated in the manifestation and treatment of mental and psychosomatic disorders, and as a result, predict therapy outcomes with accuracy.

The strengths of our study include a relatively large, well-powered and diverse sample drawn from three different studies on effective treatments of two common psychiatric disorders and one psychosomatic disorder. To control for differences in sample and treatment characteristics, we performed analyses using both the entire sample and the individual groups. Although originally designed to measure the cognitive effects of frontal lobe damage, the WCST is considered a valid and established measure of PE. We age-adjusted PE scores, and re-running analyses using self-normalized number of PE (i.e. percentage PE of total errors) did not change findings. Our study design also has some limitations. Primarily, only one measure of CF, number of perseverative errors on the WCST, was used for analyses. While we acknowledge that a composite measure calculated using results from several CF-indexing neuropsychological tests would have been preferable, past studies have reported associations between this particular measure (number of PE on the WCST) and cognitive restructuring abilities (Johnco, Wuthrich & Rapee, 2013). Second, the in-house developed, online 64-card WCST version used in the current study has not been independently validated against other measures. However, principal component analysis revealed a latent structure similar to past reports (Greve, Ingram & Bianchini, 1998) and nothing in the scoring pattern suggests abnormal functioning of our version. Third, the lack of a representative healthy comparison group limits our ability to conclude whether pre-treatment CF in our clinical samples was lower than population norms. Overall WCST performance in our sample appears to be lower than previous reports of healthy Western samples (Kohli & Kaur, 2006), yet the lack of linear associations between symptom scores and number of PE suggests that reduced CF is not an integral component of these conditions. Finally, we did not include measures of cognitive restructuring skill acquisition or utilization, which could be potential mediators in the relationship between pre-treatment CF and treatment gains.

## Conclusions

Contrary to our hypothesis, we found no generic or treatment-specific predicative power of cognitive flexibility (perseverative errors on the WCST) on patient improvement following Internet-delivered psychological treatment for social anxiety disorder, depression or tinnitus. Hence, comparably lower cognitive flexibility, as measured by the perseverative errors on the WCST, should not impede successful outcomes in Internet-delivered psychological treatments.

## Supplemental Information

10.7717/peerj.1934/supp-1 Supplemental Information 1 Principal component analysis of WCST data.

10.7717/peerj.1934/supp-2 Supplemental Information 2 Analyses syntax (R script file).

## Additional Information and Declarations

### Data Deposition

The following information was supplied regarding data availability: The raw data can only be made available on request due to restrictions of the ethical review board which did not include permission to make the data publicly available. Please contact Philip Lindner with your requests: philip.lindner@psychology.su.se.
