# Supplementary material for: Does cognitive flexibility predict treatment gains in Internet-delivered psychological treatment of social anxiety disorder, depression, or tinnitus?
Source: PeerJ. 2016 Apr 18;4:e1934. doi: 10.7717/peerj.1934 (PMC4841247; doi:10.7717/peerj.1934)
Supplement: Supplemental Information 1 [file peerj-04-1934-s001.docx]

**Supplementary Table 1. Latent structure of WCST data**

|  | Component | |
| --- | --- | --- |
|  | 1 | 2 |
| SS loadings | 3.51 | 0.32 |
| Cumulative % variance explained | 88% | 96% |
|  |  |  |
| Categories completed | 0.90 | 0.33 |
| Total correct | 0.98 | 0.05 |
| Perseverative errors | -0.88 | 0.45 |
| Total errors | -0.98 | -0.05 |

SS: Sum of Squares. Principal component analysis performed on the whole sample (n=271) using the *psych* R package and forced two-component extraction with no rotation (to aid interpretation). Note that sign is arbitrary in principal component analyses, providing it is consistent. WCST variables ‘Trials to complete first category’ and ‘Completion time’ were not included in the analysis to make it comparable with the proposed composite solution in Greve, Ingram and Bianchini (2008, Archives of Clinical Neuropsychology, 13:7).
